# Royal Westminster Ophthalmic Hospital

**Published:** 1829-04-01

**Authors:** 


### ROYAL WESTMINSTER OPHTHALMIC HOSPITAL.

There are now 23 cases of cataract attending at this hospital, some lately operated on, others in a more advanced stage of recovery, and a greater number either undergoing, or awaiting treatment or operation. In three cases the cataract is combined with glaucoma. Those students who have attended for even three months, have been able to point out the complication with the greatest ease. Mr. Guthrie has on several occasions lately described the difference between them. Where glaucoma existed he said, —"the disease usually came on about the middle period of life, from 45 to 55. In women, it generally followed the cessation of the catamenia, and was a point of great importance to be attended to at that period, inasmuch as most of the defects of sight which women laboured under about that period might be attributed to some negligence in their treatment at this time. Pain is usually felt in the head at intervals, which soon becomes fixed over the eyes, and on the side of the head, sometimes over one temple and eyebrow, sometimes on both; this is often excruciating, and with its increase the sight fails. At the end of from two to three years, the pains cease of themselves, even where no medical assistance has been sought; but not before the sight has been destroyed." It was very remarkable Mr. Guthrie said, that Nature seemed to be satisfied with the mischief done, as soon as vision was lost; the pains then ceased, and the sufferer remained in comparative comfort, save that sight was gone. During this period, several slow, gradual changes took place in the internal structure of the eye, accompanied by corresponding external appearances. The cornea became very early less brilliant, the iris slightly discolored, the sclerotica gradually changed from its natural white, to a yellowish, or even a shade of a leaden colour; tortuous vessels of a dark red colour slowly developed themselves at the circumference of the eye, spreading out their ramifications as they approached the cornea, which was frequently surrounded by them, the vessels seeming to penetrate, or in other words, to come out from within at a very little distance from it. Whilst these changes were going on, others of more importance were taking place within; the choroid coat was becoming varicose, the retina thicker and opaque, the iris irregular, the pupils fixed, irregular, and dilated; vision daily decreasing. At last the lens becomes opaque, and of a greenish color, is protruded against the iris, which is convex in appearance, and apparently changed in color as well as in structure, and is pressed forwards against the cornea; or the pupil dilated irregularly to its utmost extent, leaves scarcely a distorted ring of iris visible at the edge of the partially obscured cornea through which it is seen. The eyeballi s harder to the touch. The patient has now lost all sense of light, but is free in general from pain.

Charlotte Johnson, aged 49, lost the sight of the left eye more than two years back, and applied for advice the 20th Nov. 1828. Says she suffered great pain at first over the eye, in the eyebrow, and on the side of the head, but that she has none now, and has applied because she thinks the other eye is failing. A large cataract of a whitish color, with a slight shade of green, is immediately seen on looking at the eye; the pupil is dilated to its utmost extent so as to appear a mere ring, of an irregular form; the sclerotica is of a dull, and somewhat leaden color, some three or four large, tortuous vessels of a dark red color, run from the circumference to near the cornea, which is not clear and shining. She has no sight whatever, and cannot distinguish light from darkness. There is little alteration to be observed in the other eye, save perhaps a less degree of brilliancy than is natural to the cornea, and a little sluggishness in the motion of the pupil; the sight is diminishing. The treatment has been cupping behind the ears, a blister to the neck, and purgatives from day to day, such as the pulv. jalap. c. or the pil. hydrarg. c. gumbogia. The woman thinks her sight rather better. In another case in which the disease had commenced in both eyes it has been arrested; and in a third fully formed and combined with cataract, the pains have been removed, and the patient rendered comfortable. Mr. Guthrie said it was in the treatment of these and other unfortunate cases of a similar description, that the students should accustom themselves to examine the eye, so as to do it with sufficient firmness and gentleness; but which would be only acquired in perfection by doing it on each other. In all operations for cataract, the surgeon should do the whole without an assistant, particularly in that by extraction, and in every case, almost without an exception, the incision should be made upwards. It was equally practicable, and as easy of execution on the left as on the right eye, when the surgeon had been properly taught. Mr. Guthrie then proceeded (Tuesday, Dec. 9th) to operate on the left eye of Mrs. Fordham, affected with hard cataract. Being seated on a high chair which supported the head, he elevated the upper lid, with one finger, and fixed the eye with another, the hand being passed from behind over the head. The lower lid was pressed by the little finger of the right hand brought round in front, which also rested upon the edge of the orbit, and formed the point of support for the hand. The incision of the cornea upwards was then made by one steady equal passage of the knife through it; Mr. Guthrie stating at the same time, that he generally left a small portion of the cornea at the upper part undivided, in order to prevent any sudden spasm deranging the internal structure from the want of resistance occasioned by the whole of the upper part being too rapidly divided. The right angular hook was then introduced and the capsule torn. The lens readily came through the opening, when the eye was bound up. Little or no pain followed, there was no occasion for either bleeding or cupping, and on the fourth day, the patient was walking about, with only a shade over the eye, seeing very well.

